# Early surgical debridement in the management of infectious scleritis after pterygium excision

**DOI:** 10.1007/s12348-012-0062-1

**Published:** 2012-02-22

**Authors:** Ethan H. Tittler, Pho Nguyen, Kelly S. Rue, Daniel V. Vasconcelos-Santos, Jonathan C. Song, John A. Irvine, Ronald E. Smith, Narsing A. Rao, Samuel C. Yiu

**Affiliations:** 1 Keck School of Medicine, University of Southern California, Los Angeles, CA USA; 2 Doheny Eye Institute, Los Angeles, CA USA; 3 Department of Ophthalmology, The Wilmer Eye Institute, The Johns Hopkins University, 400 N Broadway/Smith Bldg 6001-R, Baltimore, MD 21231 USA

**Keywords:** Infectious scleritis, Pterygium excision, Surgical debridement, Biofilm

## Abstract

**Purpose:**

The purpose of this study was to report outcomes of infectious scleritis after pterygium surgery, managed with antibiotic therapies and early scleral debridement.

**Methods:**

Retrospective chart review of 13 consecutive cases of infectious scleritis after pterygium excision between 1999 and 2009 was conducted. Collected data included prior medical and surgical history, latency period between pterygium surgery and presentation of infectious scleritis, culture and histopathologic findings, antibiotic regimen, length of hospital stay, visual acuity before and after treatment, and complications.

**Results:**

Median follow-up was at 14 months. Twelve patients underwent prompt surgical debridement after infectious scleritis diagnosis (median, 2.5 days). Debridement was delayed in one patient. Median hospital stay was 3 days. Best-corrected visual acuity improved in ten patients, remained stable in one patient, and decreased in two patients following treatment. Complications included scleral thinning requiring scleral patch graft (1/13), glaucoma (3/13), and progression to phthisis bulbi (1/13). No patients required enucleation.

**Conclusions:**

In contrast to the generally poor outcomes in the literature, early surgical debridement of pterygium-associated infectious scleritis appears to offer improved prognosis.

## Introduction

Infectious scleritis is an uncommon inflammatory disease that often results in destructive complications, including loss of the affected eye [1]. Infectious scleritis can be classified as either primary, following local infection, usually after violation of scleral integrity, or secondary, from extension or dissemination of infection from another site [2–5]. Though rare, infectious scleritis is a well-documented complication of pterygium excision that may emerge as late as two to four decades postoperatively [6, 7]. Adjunctive therapies, such as β-irradiation, mitomycin C, and excessive cauterization, have been implicated in the pathogenesis of infectious scleritis after pterygium excision.

Medical management of infectious scleritis typically results in poor prognosis [4]. Surgical debridement, when performed, is often delayed, and these cases are frequently characterized by extended hospitalizations, deleterious complications, and dismal visual outcomes [6–9]. Prompt surgical debridement and appropriate antibiotic coverage have been associated with better outcomes. Here, we evaluated long-term outcomes in a cohort of patients with infectious scleritis after pterygium excision, managed with prompt surgical debridement and fortified antibiotics.

## Methods

This is a retrospective review of 13 consecutive patients diagnosed with infectious scleritis following pterygium excision and referred to the Doheny Eye Institute Cornea Service from 1999 to 2009. All pterygium excisions were performed at outside facilities. This Institutional Review Board-approved study followed the tenets of the Declaration of Helsinki; chart review was performed in accord with the Health Insurance Portability and Accountability Act of 1996.

Diagnosis of infectious scleritis was established by four corneal specialists (SCY, JCS, JAI, RES) based on presenting signs and symptoms and, in some cases, positive bacterial culture of scleral materials. Cases with negative culture results were included if there was high clinical suspicion for infectious scleritis, e.g., history of pterygium excision, presence of purulent materials, response to antibiotics, or suggestive histopathologic findings. All of our patients were referred from outside facilities, and a majority of these patients was already on topical antibiotics

Charts were reviewed, and demographic and clinical information, including age, sex, past medical and ocular history, the ophthalmologic examination, surgical procedures, treatment strategies, and outcomes, were collected and stored in an anonymous electronic database. Results of diagnostic procedures, namely culture and antibiogram of scleral material excised during surgical debridement and histopathologic examination of biopsied tissue, were also gathered and recorded similarly. Operative records from each patient's previous pterygium surgery at other centers were unavailable, but any relevant information disclosed by the patient and documented in the chart was also collected. Common descriptive statistical methods (mean, median, standard deviation) were used for data analysis.

### Technique of scleral debridement

All patients received general anesthesia, followed by preparation of the affected eye with 5% povidone–iodine solution and standard sterile technique. After placement of a wire lid speculum, the conjunctiva was approached superiorly with a peritomy, using Westcott scissors. The necrotic tissue and abscess materials were dissected under stereotactic microscopy with a Beaver 66 surgical blade and scissors, until the choroidal pigmentation was visible through the scleral fibers. No globe penetration occurred in any case. In cases that involved the sclera beneath the rectus muscle, the muscle was detached and re-anchored with interrupted 8-0 Vicryl sutures to healthy sclera after complete debulking of the underlying necrotic tissue. Occasional gentle hemostasis was achieved using bipolar cautery. After debridement, the surgical defect was left exposed, without primary closure, to ensure good exposure to the topical fortified antibiotics. In one case (patient 3), an amniotic membrane graft was used. The conjunctival edge was tucked down and anchored with interrupted 8-0 Vicryl sutures. Subconjunctival injection of vancomycin (14 mg/mL) and tobramycin (25 mg/mL) was performed. Bacitracin ointment was dispensed over the ocular surface, and the eye was protected with a light-pressure eye patch and shield for 24 h.

## Results

A total of 13 patients were included in the study (Table 1). The median age at presentation was 68 years (mean, 69.5 years; range, 49–92 years). Seven (54%) patients were female, and six (46%), male. Past medical history was significant for diabetes mellitus in five patients (38.5%). Two patients reported adjunctive mitomycin C treatment, and one reported β-irradiation treatment at the time of pterygium resection. Elapsed time between pterygium excision and onset of infectious scleritis ranged from 4 months to 20 years. All patients were referred to our tertiary care center; ten patients already had received treatment from the referring physicians, such as topical and oral antibiotics, with or without corticosteroids.

**Table 1:** Clinical features of a series of patients with infectious scleritis after pterygium surgery

Patient	Age at presentation, sex	Pterygium adjunctive therapy	Previous therapy	Latency period^a^ (years)	Other presenting findings	Relevant medical history	Bacterioscopy/culture result	Histopathology of scleral tissue	Time between presentation and debridement (days)
1	73, F	β-Irradiation	Tobramycin/dexamethasone gtts, ciprofloxacin ointment, glue	8	Serous RD/choroidal effusion, flare	Rheumatoid arthritis	*Pseudomonas* sp.	Acute/chronic necrotizing scleritis^c^	15, 23
2	61, F	N/A	–	N/A	–	–	*Pseudomonas* sp.	Acute/chronic necrotizing scleritis^c^	0
3	79, M	N/A	–	20	–	DM	No growth	Acute/chronic necrotizing scleritis^c^	1
4	72, M	N/A	Erythromycin ointment	1	Choroidal effusion, posterior synechiae	Alcoholism	No growth	Acute/chronic necrotizing inflammation, fibrotic material within blood vessels, calcified material^c^	3
5	83, F	Mitomycin C	Ofloxacin gtts, moxifloxacin gtts	0.33	NVI	–	*Pseudomonas* sp.	Acute/chronic necrotizing scleritis	5
6	64, M	N/A	Diclofenac gtts, rimexolone gtts, prednisolone gtts, homatropine gtts	3	Choroidal effusion	DM	*Eikenella corrodens*	Acute necrotizing scleritis^c^	2
7	92, F	N/A	Moxifloxacin gtts, gatifloxacin gtts, doxycyclin PO, moxifloxacin PO	N/A	Meibomian gland dysfunction	DM	No growth	Acute necrotizing inflammation	16
8	64, F	Mitomycin C	Moxifloxacin gtts, erythromycin gtts	1.5	Serous RD, angle-closure	DM	*Pseudomonas* sp.	Acute/chronic necrotizing scleritis	14
9	49, M	N/A	Ofloxacin gtts	3	Glaucoma	DM	*Streptococcus viridans* in thio broth only	Chronic inflammation	0, 28
10	68, F	N/A	–	N/A	Perforation	Rheumatoid arthritis	*Staphylococcus aureus*, one colony	Necrotizing scleritis^c^	18
11	65, F	N/A	Tobramycin/dexamethasone gtts, homatropine gtts	12	Choroidal detachments	–	*Pseudomonas* sp.	Acute/chronic necrotizing scleritis^c^	2
12	56, M	N/A	Removal of calcium plaque over pterygium resection site, fluorometholone gtts, prednisolone, neomycin/polymyxin B/dexamethasone ointment	4	Dry eye	–	*Pseudomonas* sp.	–	32
13	77, M	–	Prednisolone gtts, neomycin/polymyxin B/dexamethasone ointment, cyclopentolate, atropine, brimonidine	3	RD	–	*Pseudomonas* sp.	–	1
Averages	69.5			5.9^b^					6.4

*gtts* drops, *RD* retinal detachment, *N/A* not available, *DM* diabetes mellitus, *NVI* iris neovascularization

^a^Time elapsed between pterygium excision and diagnosis of infectious scleritis

^b^Not including N/A data

^c^Negative special stains for bacteria (Gram) and fungi (Gomori methenamine silver)

Once the patients arrived at our institution, 12 out of 13 underwent prompt surgical debridement. For the remaining patient, debridement was delayed for 32 days because of an unusual calcific plaque overlying the affected area and an initial dramatic improvement on topical fortified antibiotics alone. Excluding this outlier, the median length of time between presentation and debridement was 2.5 days (range, 0–18 days).

Cultures of scleral scrapings were positive for *Pseudomonas* sp. in seven cases, *Eikenella corrodens* in one patient (6), *Streptococcus viridans* in one patient (9), and *Staphylococcus aureus* in one patient (10). Histopathological reports were available for 11 of the 13 patients, revealing acute suppurative inflammation in ten cases and chronic inflammatory infiltrate in eight cases. Special stains for bacteria (Gram) and fungi (Gomori methenamine silver) were documented as negative in seven specimens.

All patients received antibiotic therapy (Table 2) in addition to surgical debridement. In all cases, patients initially received fortified topical eye drops every hour for several days. Tobramycin was used in all cases, in combination with vancomycin, cefazolin, and/or piperacillin. Eleven patients also received systemic IV and/or oral antibiotics. The use of systemic antibiotics was guided by each patient's clinical presentation and course. The antibiotic regimens were adjusted according to clinical course and availability of culture and antibiogram.

**Table 2:** Treatment outcomes after debridement and antibiotic therapy for infectious scleritis

Patient	Hospital stay (days)	Complications	Antibiotic therapy	BCVA before debridement	BCVA after debridement	Follow-up (months)
1	21	Bleeding, scleral thinning, glaucoma, recurrence	Ciprofloxacin IV, gentamicin IV, tobramycin/dexamethasone gtts, ciprofloxacin ointment, fortified topical cefazolin and tobramycin and vancomycin and piperacillin gtts, ciprofloxacin ointment, ciprofloxacin PO	HM	20/50 −2	86
2	2	Severe scleral thinning, involvement of sclera at medial rectus	Levofloxacin IV, tobramycin/dexamethasone gtts, moxifloxacin gtts, gatifloxacin gtts	20/100	20/40	30
3	1	Scleral thinning, involvement of sclera at medial rectus	Fortified topical vancomycin and tobramycin gtts, gatifloxacin gtts, ciprofloxacin PO	20/200	20/50	4
4	1	Scleral thinning	Levofloxacin IV, fortified topical vancomycin and tobramycin gtts, gatifloxacin gtts, ciprofloxacin ointment	20/100 −1	20/40 +2	14
5	3	Scleral thinning, entropion, posterior synechiae	Cefazolin IV, fortified topical vancomycin and tobramycin gtts, ciprofloxacin gtts, moxifloxacin gtts	LP	CF	4
6	2	Severe scleral thinning requiring scleral patch graft and patch graft revision, involvement of sclera at medial rectus	Fortified topical vancomycin and tobramycin gtts, gatifloxacin gtts, gentamicin ointment, levofloxacin PO	20/100	20/50 +1	5
7	1	Scleral thinning, corneal necrosis with iris adhesion, prolapsed vitreous, elevated IOP	Levofloxacin IV, Fortified topical vancomycin and tobramycin gtts, gatifloxacin gtts, moxifloxacin gtts, moxifloxacin PO, fluconazole PO, doxycyclin PO	LP	LP	4
8	7	Severe scleral thinning, glaucoma	Gentamicin IV, ciprofloxacin IV, fortified topical tobramycin and vancomycin gtts, moxifloxacin gtts, loteprednol/tobramycin gtts, ciprofloxacin ointment, ciprofloxacin PO	3/200	20/30 −2	26
9	5, 1	Scleral thinning, recurrence	Fortified topical vancomycin and tobramycin gtts, moxifloxacin gtts, gatifloxacin gtts, ofloxacin gtts, ciprofloxacin gtts	20/200	HM	44
10	0	Scleral thinning, perforation, corneal haze, corneal epithelial defect that resolved	Fortified topical tobramycin and cefazolin gtts, ofloxacin gtts	20/50	20/25 +1	62
11	14	Scleral thinning, choroidal detachment, involvement of sclera under medial rectus	Ciprofloxacin IV, fortified topical tobramycin and piperacillin gtts, tobramycin/dexamethasone gtts, levofloxacin gtts, ofloxacin gtts	20/80 +2	20/60 +1	6
12	4	Scleral thinning, hypertrophic conjunctival growth, choroidal detachments, serous retinal detachment	Ciprofloxacin IV, fortified topical tobramycin gtts, levofloxacin gtts	20/25	20/20	13
13	10	Scleral thinning, phthisis	Fortified topical tobramycin and vancomycin gtts, levofloxacin gtts, ciprofloxacin ointment, ciprofloxacin PO, erythromycin gtts	CF @ 5′	NLP	102

*gtts* eye drops, *IOP* intraocular pressure, *BCVA* best-corrected visual acuity, *LP* light perception, *CF* counting finger, *HM* hand motion, *NLP* no light perception

Two patients (1 and 10, Table 1) presented with history of rheumatoid arthritis and pterygium excision, which complicated the diagnostic algorithm. Nonetheless, the scleral scrape cultures were positive for bacteria in both. The presenting clinical features of patient 1 were especially complicated, including severe intraocular inflammation and choroidal and retinal double detachments. She ultimately required a second surgical debridement, 8 days after the first, with excellent visual restoration. Patient 9 also required a second debridement, 28 days after the first. Patient 8 presented with the clinical picture of endophthalmitis and angle-closure glaucoma, so surgical intervention was delayed 14 days, until the diagnosis of infectious scleritis was confirmed. Overall, the median hospital stay was 3 days (mean, 6.7 days; range, 0 to 21 days).

**Fig. 1:**
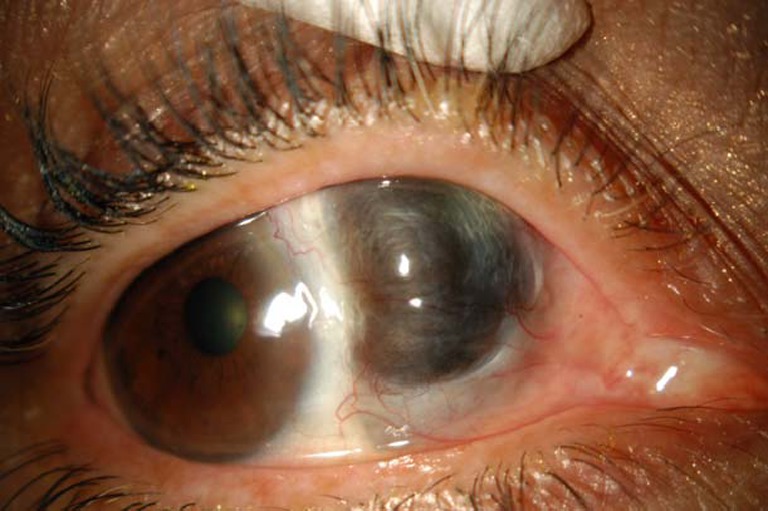
Severe thinning of the sclera following debridement of infectious scleritis

Globe preservation was achieved in all 13 eyes, despite scleral thinning in each case (Table 2 and Fig. 1). There was documented necrosis of the sclera under the medial rectus in four patients, requiring the muscle to be detached for debridement. The medial rectus was then transfixed to the adjacent healthy sclera using interrupted 8-0 Vicryl sutures. Over the follow-up period (mean follow-up of 11.25 months), these patients did not complain of diplopia despite good visual rehabilitation (20/40 to 20/60).

Preoperative best-corrected visual acuity (BCVA) ranged from 20/25 to light perception (Table 2). Postoperative BCVA ranged from 20/20 to no light perception (patient 13). Ten patients demonstrated improvement in their BCVA; one patient's BCVA remained unchanged. Two patients (9 and 13), whose preoperative visual acuities of 20/200 and counting fingers at 5 ft, respectively, decreased to hand motion and no light perception, respectively, at last visit.

## Discussion

We report improved outcomes of infectious scleritis after pterygium excision managed with prompt, aggressive scleral debridement and antibiotic therapy, and we contend that delayed debridement may carry worse outcomes. None of the patients in our series required enucleation; 11 of the 13 patients demonstrated stable or improved visual acuity, and the median hospital stay of our patients was 3 days. Other investigators have demonstrated higher complication rates, poorer visual outcomes, and longer hospital stays for the treatment of infectious scleritis.

In one review of 28 patients with culture-proven infectious keratoscleritis, 88% of patients who initially underwent medical management by topical, intravenous, or subconjunctival fortified antibiotic regimens eventually required evisceration (Table 3) [4]. While surgical intervention combined with antibiotics was associated with improved outcomes, enucleation was still necessary in 18% of these cases. Lin et al. [6] reported an average hospitalization of 32.5 days, and 62% of their patients required repeated debridements. Hsiao et al. [7] reported various complications: serous retinal detachment (11%), choroidal detachment (17%), double detachment (11%), complicated cataracts (28%), recurrence of initial infection (22%), and eventual evisceration (22%). Only 61% of their patients regained some useful vision; all were initially treated with antibiotics, and 78% eventually required operative management. In one retrospective study of 16 infectious scleritis cases over a 10-year period, nine patients received only medical management; 22% of these patients eventually required enucleation, and only 22% attained a BCVA of better than 2/200, with an average hospitalization of 21.2 ± 4.8 days [8]. A more recent study reported the application of subpalpebral antibiotic lavage in six patients who had demonstrated no clinical improvement while on topical fortified antibiotics [9]. All six patients had resolution of infectious keratoscleritis; unfortunately, more than half of these patients required corneal transplant, cataract extraction, glaucoma aqueous shunt, or some combination of these surgical treatments. In a series of three cases of post-pterygium excision *Pseudomonas sp*. keratoscleritis, Huang and colleagues reported the use of intensive topical and systemic antibiotics, low-dose oral prednisolone, and early surgical debridement [10]. There were no cases of recurrence or evisceration; however, the BCVA remained suboptimal, ranging from 20/120 to 20/400. The lower threshold for surgical intervention in this current study appears to correlate with better postoperative BCVA, 100% globe preservation rate, fewer complications, and shorter hospital stays when compared to the literature.

**Table 3:** A comparison of treatment outcomes of infectious scleritis reported in other studies

Study	Number of patients	At least one debridement	Repeat debridement	Postoperative BCVA (%)	Mean hospital stay (days)	Postoperative complications (%)	Enucleations or eviscerations
Reynolds and Alfonso [4]	28	39%	N/A	N/A	N/A	N/A	32%
Lin et al. [6]	30	87%	62%	N/A	32.5	N/A	3.3%
Hsiao et al. [7]	18	33%	N/A	NLP (22)	N/A	Recurrence (22)	22%
				LP (5.5)		Complicated cataract (28)	
				HM (5.5)		Serous retinal detachment (22)	
				CF (5.5)			
				20/400 (5.5)		Choroidal detachment (17)	
				20/200 (28)			
				20/100 (11)		Choroidal and retinal detachment simultaneously (11)	
				20/50 (11)			
				20/30 (5.5)			
Huang et al. [10]	3	100%	0%	20/200 (67)	N/A	Posterior synechiae (67)	0%
				20/120 (33)		Cataract (67)	
						Cystoid macular edema (67)	
Huang et al. [8]	16	44%	13%	NLP (19)	N/A	Vitreous opacity (50)	19%
				LP (19)		Glaucoma (25)	
				HM (6)		Serous retinal or choroidal detachments (25)	
				0.02 (6)			
				0.1 (13)			
				0.2 (6)		Cataract (13)	
				0.3 (6)		Recurrence (13)	
				0.5 (6)			
				0.6 (6)			
				N/A (13)			

*BCVA* best-corrected visual acuity, *N/A* not available, *NLP* no light perception, *LP* light perception, *HM* hand motion

As previously reported, *Pseudomonas* sp. was the most commonly isolated organism in our series. *Pseudomonas* has been associated with more aggressive scleral infection and a dismal prognosis [4]. Other organisms are tabulated in Table 1. Interestingly, *Streptococcus viridans* and *Staphylococcus aureus* were isolated from patients 9 and 10, respectively. It is unclear if these organisms represented the true infectious agents or contaminations from normal flora. *E. corrodens*, a gram-negative rod rarely recovered in soft tissue infections, was isolated from patient 6 [11].

Even though no microorganisms were observed on histopathologic evaluation of the scleral specimens, most revealed acute, necrotizing (suppurative) inflammation, and many had a mixed acute–chronic infiltrate consistent with bacterial infections scleritis [12]. A possible explanation would be administration of topical antibiotics by the referring physicians and implementation of aggressive antibiotic regimen prior to surgical debridement at our institution.

During debridement, the surgeons noted small pockets of abscess within the necrotic sclera and beneath areas of seemingly healthy sclera, consistent with two previous reports [6, 12]. All of the patients in this series had a history of pterygium excision. The latency period between pterygium resection and scleral infection in our series, 4 months to 20 years, is comparable to the literature [6]. Application of adjunctive therapies such as mitomycin C and b-radiation at the time of pterygium has been implicated in the pathogenesis of scleral infection through destruction of the protective blood supply to the conjunctiva and outer sclera. Diabetes mellitus, another possible risk factor predisposing patients to infectious scleritis [13], was present in five (38%) of our cases.

Biofilm formation may also be implicated in the refractory nature of infectious scleritis to medical management alone. In the human body, bacteria exist predominantly in two states: planktonic and sessile [14]. Planktonic forms are highly susceptible to antibiotics and host defenses, whereas sessile forms are more resistant. The sessile form is seen mainly as a biofilm on the surfaces of implanted devices or biologic tissues. Within the biofilm architecture, bacterial colonies are encased in an extracellular matrix of exopolysaccharides, which provide protection from hostile environments. The inner, sessile, oxygen-deprived microcolonies have limited mobility, a slow growth rate, a different synthetic rate, and a reduced susceptibility to antibiotics [15]. For some antibiotics, the concentration required to penetrate the biofilm can be thousands of times the concentration required for planktonic bacteria of the same strain [14, 16, 17]. The biofilms may evolve into a physically diverse structure containing channels and interstitial voids, as previously reported [12]. Thus, physiological heterogeneity within a bacterial colony and pharmacokinetic limitations are possible explanations for the frequent failure of antibiotic treatment alone in the management of infectious scleritis [18–20]. Accordingly, early surgical debridement may be important in disrupting the barriers to antibiotic efficacy. This was our rationale for a lower threshold for surgical debridement in this study.

After debridement, we decided against the use of cryopreserved human amniotic membrane graft on the exposed, debrided area in all but one patient (3). Maximum exposure is desirable for topical antibiotic penetration and prevention of incubation of any microbes remaining at the surgical site. Hydration of the bare and thinned sclera with continual medical therapy has been suggested as an effective modality to facilitate re-epithelialization of the sclera after bare sclera excision of pterygium [21]. Second, we wanted to avoid obscuring visualization of early recurrence or the formation of abscesses [6, 22]. Adequate re-epithelialization of the exposed sclera was observed in 12 eyes shortly after debridement. For patient 3, however, a human amniotic membrane graft was deemed appropriate because re-epithelialization did not appear likely to occur due to the severity of scleral thinning [21].

As previously discussed, none of the patients with involvement of the sclera under the medial rectus complained of diplopia following surgery, possibly indicating that they were able to achieve fusion or that there was suppression of the misaligned eye. Management of diplopia would involve prism or strabismus surgery. However, surgical management may not be a feasible option given the poor structural integrity of the debrided eye (Fig. 1).

Thus, prompt and aggressive surgical debridement may contribute to better outcomes in infectious scleritis after pterygium excision. We postulate that the generally poor outcomes reported in the literature may be related to the limited penetration of antibiotics into bacterial microabscesses and biofilms in the nearly avascular, infected sclera and to the delay in surgical intervention aimed at disrupting these defenses and debulking the microbial load. Prospective, comparative studies may not be possible because this disease entity has low incidence and high risks of permanent vision impairment or loss of globe. As other studies reported high rates of conversion to surgical debridement [4, 6–8], we advocate vigilant monitoring and prompt debridement if indicated.
